# Higher valency vaccines’ impact on antimicrobial resistance rates in *Streptococcus pneumoniae* causing invasive disease: a retrospective analysis based on national reference laboratory data, Belgium, 2018 to 2023

**DOI:** 10.2807/1560-7917.ES.2025.30.45.2500179

**Published:** 2025-11-13

**Authors:** Helene Vermeulen, Lize Cuypers, Boudewijn Catry, Stefanie Desmet, Niel Hens

**Affiliations:** 1 Interuniversity Institute for Biostatistics and statistical Bioinformatics (I-BIOSTAT), Data Science Institute (DSI), Hasselt University, Hasselt, Belgium; 2 National Reference Centre for Invasive Pneumococci, Department of Laboratory Medicine, University Hospitals Leuven, Leuven, Belgium; 3 Laboratory of Clinical Microbiology, Department of Microbiology, Immunology and Transplantation, KU Leuven, Leuven, Belgium; 4 Department of Epidemiology and public health, Sciensano, Brussels, Belgium; 5 Faculty of Medicine, Université libre de Bruxelles (ULB), Brussels, Belgium; 6 Centre for Health Economic Research and Modelling Infectious Diseases (CHERMID), Vaccine & Infectious Disease Institute (VAXINFECTIO), University of Antwerp, Antwerp, Belgium

**Keywords:** *Streptococcus pneumoniae*, antibiotic use, pneumococcal conjugate vaccines, antimicrobial resistance, modelling, statistics

## Abstract

**BACKGROUND:**

Fifteen- and 20-valent pneumococcal conjugate vaccines (PCVs) offer broader protection against invasive pneumococcal disease (IPD) than PCV13. Adopting these vaccines may result in decreasing IPD incidence, antibiotic use and antimicrobial resistance (AMR) rates. If the additional serotypes in PCV15 and PCV20 are associated with AMR, AMR rate reduction could be greater than expected from reduced antibiotic consumption alone.

**AIM:**

This retrospective analysis assessed the association between AMR and non-PCV13 serotypes in PCV15 and PCV20.

**METHODS:**

Laboratory-based surveillance data on 8,123 IPD isolates were obtained retrospectively from the Belgian Reference Centre for invasive *Streptococcus pneumoniae*. Isolates (n = 8,088) were serotyped and tested for AMR. Associations between vaccine serotype groups and AMR were evaluated by multinomial logistic regression. Where associations varied with patients’ age, ranges of odds ratios (ORs) are presented.

**RESULTS:**

PCV15-non-PCV13 and PCV20-non-PCV13 serotypes accounted for 7.4% (n = 597) and 37% (n = 2,992) of IPD isolates respectively. Of non-PCV20 serotypes, 24% (508/2,125) were penicillin resistant. Compared with non-PCV20 serotypes, PCV15-non-PCV13 serotypes were more often associated with erythromycin (ORs: 3.59–9.43) and tetracycline (OR: 2.00) resistance, and with trimethoprim/sulfamethoxazole (OR: 0.11) susceptibility. PCV20-non-PCV15 serotypes were more often associated with amoxicillin (OR: 9.45) and cefotaxime (ORs: 5.06–82.38) resistance, and with erythromycin (ORs: 0.13–0.18), tetracycline (OR: 0.71) and penicillin (ORs: 0.05–0.46) susceptibility.

**CONCLUSION:**

PCV20 may lead to a larger decrease in overall IPD incidence than PCV15. Although the PCV20 vaccination impact on AMR may be limited, some resistant or difficult to treat infections could be avoided. Serotype replacement might lead to infections with low level penicillin resistance increasing, but most of these should remain treatable.

**Key public health message**

**What did you want to address in this study and why?**

Infections by pneumococci can cause invasive pneumococcal disease (IPD). Pneumococcal conjugate vaccines (PCVs) prevent infections by pneumococci of certain serotypes and may also address antimicrobial resistance (AMR) in pneumococci by reducing antibiotic use and incidence of serotypes associated with AMR. PCV13, covering 13 serotypes, is currently used in Belgium. We studied if additional serotypes in new available PCVs were AMR associated.

**What have we learnt from this study?**

We investigated 8,123 IPD isolates from Belgium. We found that 7.4–37.0% of the pneumococcal serotypes under study were covered by the new PCVs and not by PCV13. Compared with serotypes not covered by any PCVs, serotypes covered by one of the new PCVs (i.e. PCV20) but not by the current PCV13, showed higher amoxicillin and cefotaxime resistance rates, but lower erythromycin, tetracycline and penicillin resistance rates.

**What are the implications of your findings for public health?**

In Belgium, implementation of the new PCVs may lead to a decrease in overall IPD incidence and might avoid some resistant and otherwise difficult to treat pneumococcal infections. On the other hand, serotype replacement might lead to an increase in the incidence of serotypes that are not included in any of the PCVs, which could in turn lead to a relative increase in AMR rates in pneumococci, although most of these infections should remain treatable.

## Introduction

*Streptococcus pneumoniae* (pneumococcus) is a commensal Gram-positive bacterium that colonises the upper respiratory tract. In children, older adults and immunocompromised individuals, *S. pneumoniae* can opportunistically cause invasive pneumococcal disease (IPD), including clinical manifestations as pneumonia, bacteraemia and meningitis. Based on the polysaccharide capsule of the pneumococcus, more than 100 different serotypes have been described [1]. Over time, several pneumococcal conjugate vaccines (PCVs) have become available, including PCV7, PCV10, PCV13, PCV15 and PCV20. Each of these vaccines offer protection against IPD, while targeting a different number of serotypes [2]. In Belgium, universal vaccination of infants under 2 years of age with PCV7, covering seven serotypes, was introduced in the national childhood immunisation programme in 2007 and quickly achieved high uptake (> 90%) [3-6]. In 2011, a switch to PCV13 (13 serotypes) was made. In 2015 in Flanders and in 2016 in Wallonia, the Belgian government decided to switch from PCV13 to the lower-valent PCV10, as both were deemed equally effective [7,8]. Soon after, an increase in serotype 19A (a PCV13-non-PCV10 serotype) was observed and it was decided to switch back to PCV13 in 2019, which has been used ever since [9]. For adults aged above 65 years, individuals with comorbidities and immunocompromised, vaccination against IPD is also recommended, although vaccine uptake remains much lower in this group (18–32%) [10].

After the introduction of PCV7 in Belgium, the incidence of IPD caused by PCV7 serotypes in children decreased significantly. However, due to serotype replacement, the incidence of non-PCV7 serotypes increased. Similarly, after the first introduction of PCV13, a steep decline in PCV13 serotypes was observed, accompanied by an increase in non-PCV13 serotypes [11]. Because of the issue of serotype replacement, new pneumococcal vaccines covering more serotypes and providing broader protection against IPD are being investigated. In 2021 and 2024, PCV15 and PCV20 received approval from the European Medicines Agency (EMA) for their use in children, protecting against two and seven additional serotypes, respectively, compared with PCV13 [12-14]. In 2023, the Belgian Superior Health Council declared that PCV13 and PCV15 are equally effective in the vaccination of children and adolescents for the prevention of IPD [15]. The serotypes included in each of these pneumococcal vaccines are shown in Table 1. Although serotype 6C is not included in PCV13, PCV15 and PCV20, it is often considered a PCV-related serotype due to the cross-protection that these three vaccines offer by including serotype 6A [16].

**Table 1:** Serotypes included in pneumococcal conjugate vaccines (n = 20 serotypes)

Vaccine	Serotypes included
PCV7	4, 6B, 9V, 14, 18C, 19F, 23F
PCV10	4, 6B, 9V, 14, 18C, 19F, 23F, 1, 5, 7F
PCV13	4, 6B, 9V, 14, 18C, 19F, 23F, 1, 5, 7F, 3, 6A, 6C^a^, 19A
PCV15	4, 6B, 9V, 14, 18C, 19F, 23F, 1, 5, 7F, 3, 6A, 6C^a^, 19A, 22F, 33F
PCV20	4, 6B, 9V, 14, 18C, 19F, 23F, 1, 5, 7F, 3, 6A, 6C^a^, 19A, 22F, 33F, 8, 10A, 11A, 12F, 15B

PCV: pneumococcal conjugate vaccines.

**^a^** Not included in the PCV but considered a PCV-related serotype due to cross-protection of serotype 6A [16].

Pneumococcal vaccines are not only useful in preventing IPD but may also address antimicrobial resistance (AMR) in pneumococci by reducing antibiotic use [17]. Both antibiotic community consumption, and AMR in pneumococci are relatively high in Belgium compared with the European average [18]. However, the difference in penicillin resistance observed between Belgium and other countries is mainly due to the use of a lower penicillin breakpoint to define resistance [18]. Several studies have reported reduced resistance rates in *S. pneumoniae* after PCV introduction [19-21]. In Belgium, after the first introduction of PCV13, a decrease in resistance rates to penicillin and erythromycin was observed in strains affecting infants younger than 2 years. After the switch to PCV10, resistance rates to both antibiotics increased again [11]. As PCV15 and PCV20 offer broader protection against IPD compared with PCV13, use of these vaccines may prevent pneumococcal disease, which might result in decreased incidence of antibiotic treatments and lower AMR rates in *S. pneumoniae*. Furthermore, if the additional non-PCV13 serotypes included in PCV15 and PCV20 appear to be associated with AMR in the current epidemiological landscape, the potential reduction in AMR could be even greater than expected from the reduction in antimicrobial consumption alone [22]. We therefore assessed the association between AMR and the additional non-PCV13 serotypes in PCV15 and PCV20 in Belgium over a 6-year observation period (2018–2023).

## Methods

### Data collection and microbiological analysis

For this retrospective analysis, laboratory-based surveillance data on IPD isolates collected from normally sterile sites were obtained from the Belgian National Reference Centre (NRC) for invasive *S. pneumoniae* [23]. The NRC has organised voluntary laboratory-based surveillance in Belgium for over 25 years. All clinical laboratories in Belgium are invited to send IPD isolates to the NRC, hosted at the University hospital in Leuven. The number of laboratories sending IPD isolates has remained stable over time, with around 90 laboratories participating each year [24]. While strains derived from all clinical manifestations of IPD are collected at the NRC, 94% was isolated from blood, 4% from cerebrospinal fluid and 2% from other normally sterile body fluids. As only a few laboratories in Belgium do not participate in IPD surveillance, coverage is estimated to exceed 92% for the years 2018–2023.

The NRC performs antimicrobial susceptibility testing and determines the pneumococcal serotype for all IPD isolates received. Isolates were serotyped by the Quellung reaction with serotype-specific sera (SSI Diagnostica, Hillerød, Denmark) and classified as either PCV13-related serotype (including serotype 6C), PCV15-non-PCV13 serotype, PCV20-non-PCV15 serotype or non-PCV20 serotype (Table 1). Since typing to serotype level was not routinely performed for all isolates before 2018, data from before 2018 were excluded from the analysis.

Disk diffusion was used to assess AMR to oxacillin, erythromycin, tetracycline and trimethoprim/sulfamethoxazole antibiotics, according to the incubation condition guidelines and interpretation criteria of the European Committee of Antimicrobial Susceptibility Testing (EUCAST) [25,26]. In case of reduced β-lactam susceptibility according to the disk diffusion method (oxacillin zone diameter < 20 mm), the minimum inhibitory concentration (MIC) for penicillin, amoxicillin and cefotaxime was measured by Etest (BioMérieux, France) until July 2020, or by the Sensititre broth microdilution method afterwards. Isolates with a penicillin MIC value higher than 0.06 mg/L or an amoxicillin MIC value higher than 0.5 mg/L were considered resistant, according to the EUCAST clinical meningitis breakpoints [27].

All isolates were then classified as either susceptible or resistant, for each of the antibiotics tested, with the ‘I’ category considered to be susceptible. Furthermore, isolates were also categorised as resistant to at least one antibiotic, and as multidrug resistant (MDR) in case of resistance to three or more antibiotic groups (anatomical therapeutic chemical (ATC) classification level 3). In case of β-lactam resistance, the level of resistance was considered low if the penicillin MIC value was higher than 0.06 mg/L, but the amoxicillin MIC value was lower than 0.5 mg/L, while an amoxicillin MIC value higher than 0.5 mg/L was considered a high level of resistance. Information on confounding variables was obtained from the laboratory referral forms sent to the NRC.

### Variable types and categories for the analysis

The following variables were considered confounders for the association of serotype with AMR and are discussed in Table 2: year of isolation, vaccination period in Belgium, IPD diagnosis and age group of the patient.

**Table 2:** Variables considered for descriptive and statistical analysis: variable type and categories (n = 13 variables)

Variable	Variable type	Categories
Serotype group	Outcome variable	- Non-PCV20 serotypes - PCV13-related serotypes - PCV15-non-PCV13 serotypes - PCV20-non-PCV15 serotypes
Penicillin resistance	Predictor variable	- Resistant - Susceptible
Amoxicillin resistance	Predictor variable	- Resistant - Susceptible
Cefotaxime resistance	Predictor variable	- Resistant - Susceptible
Erythromycin resistance	Predictor variable	- Resistant - Susceptible
Tetracycline resistance	Predictor variable	- Resistant - Susceptible
Trimethoprim/sulfamethoxazole resistance	Predictor variable	- Resistant - Susceptible
Year of isolation	Confounder: Over time, the trend for specific serotype groups can change.	NA
Vaccination period	Confounder: The vaccine used during a specific period can have an impact on the distribution of serotype groups.	- PCV10 vaccination period (2018–2019) - PCV13 vaccination period (2020–2023)
IPD diagnosis	Confounder: Certain IPDs can be associated with certain serotype groups.	- Bacteraemia - Bacteraemia with pneumonia - Meningitis - Another clinical manifestation of IPD
Age group	Confounder: Certain age groups can be associated with certain serotype groups. The categorisation for age group is based on the vaccination schedules used in Belgium, where individuals < 2 years are routinely vaccinated, and vaccination is advised for individuals > 65 years. We further assume vaccination in infancy offers some protection during the coming years.	- < 2 years - 2–15 years - 16–65 years - > 65 years
Antibiotic resistance^a^	Descriptive variable	- Resistant - Susceptible
Multidrug resistance^b^	Descriptive variable	- Resistant - Susceptible

ATC: anatomical therapeutic chemical classification; IPD: invasive pneumococcal disease; NA: not applicable, since the year of isolation is included as a continuous variable.

^a^ Antibiotic resistance: resistance to at least one antibiotic.

^b^ Multidrug resistance: resistance to three or more antibiotic groups (ATC level 3).

### Statistical analysis

We applied descriptive statistics and performed multinomial logistic regression analyses to assess the association between vaccine serotype group and resistance to each of the antibiotics tested, while adjusting for the confounding variables (Table 2). It was assumed that the association between vaccine serotype group and antibiotic resistance might differ for different age groups, due to distinct antibiotic consumption patterns. Therefore, the interactions between age group and resistance to antibiotics were included in the analysis. Furthermore, since different vaccination periods show different trends in serotype distributions, the interaction between vaccination period and year of isolation was also included in the analysis. IPD diagnosis was included as a confounder in the analysis but did not contribute to any interaction terms. Information on the age group was missing for four of 8,088 isolates, which were excluded from the analyses.

In case resistance to different antibiotics frequently occurred together within one sample, these antibiotics were not simultaneously included as predictor variables into the models. This correlation between the resistance patterns of different antibiotics was evaluated by the phi coefficient, where a value above 0.5 was considered to indicate a strong correlation. Nonsignificant variables and interactions were excluded from the model according to a backward model selection procedure using Akaike’s Information Criterion (AIC). Statistical analyses were performed in RStudio version 4.2.2 [28] and a significance level of 5% was used.

### Study reporting

This study is reported in accordance with the guidelines of Strengthening the reporting of observational studies in Epidemiology (STROBE).

## Results

A total of 8,123 isolates were analysed from 2018 to 2023, of which 8,088 could be classified by serotype. In 2020 and 2021, coinciding with the COVID-19 pandemic, the number of isolates almost halved compared with 2018 and 2019 (884 and 845 compared with 1,558 and 1,582, respectively). The number of pneumococcal isolates increased again in 2022 (1,477) and even rose above pre-pandemic levels in 2023 (1,742). Most of the isolates under study originated from individuals aged over 65 years (3,718; 46.0%). Individuals aged 2 to 15 years contributed the fewest isolates (477; 5.9%). A total of 687 isolates (8.5%) originated from individuals below the age of 2 years and 3,202 isolates (39.6%) came from individuals aged 16 to 65 years. Table 3 shows the number and percentage of isolates by year, PCV period, serotype group, age group and diagnosis. As can be seen from Table 4, individuals below the age of 2 years showed the highest percentage of isolates resistant to at least one antibiotic and resistant to multiple antibiotics (39.7% and 16.9%, respectively), whereas individuals aged 16 to 65 years showed the lowest percentage (21.3% and 5.5%, respectively).

**Table 3:** Number and percentage of isolates by year, PCV period, serotype group, age group and diagnosis, Belgium, 2018−2023 (n = 8,088 isolates)^a^

Variable	Number	%
**Year**		
2018	1,558	19.3
2019	1,582	19.6
2020	884	10.9
2021	845	10.4
2022	1,477	18.3
2023	1,742	21.5
**PCV period**		
PCV10 period (2018–2019)	3,140	38.8
PCV13 period (2020–2023)	4,948	61.2
**Serotype group**		
Non-PCV20	2,125	26.3
PCV13-related	2,971	36.7
PCV15-non-PCV13	597	7.4
PCV20-non-PCV15	2,395	29.6
**Age group**		
< 2 years	687	8.5
2–15 years	477	5.9
16–65 years	3,202	39.6
> 65 years	3,718	46.0
Unknown	4	0.0
**Diagnosis**		
Bacteraemia	2,839	35.1
Bacteraemia with pneumonia	4,289	53.0
Meningitis	529	6.5
Other clinical manifestation of IPD	382	4.7
Unknown clinical manifestation of IPD	49	0.6

PCV: pneumococcal conjugate vaccine; IPD: invasive pneumococcal disease.

^a^ Among 8,123 isolates that were obtained between 2018 and 2023, 8,088 could be classified by serotype.

**Table 4:** Percentage of isolates resistant to at least one antibiotic and percentage of isolates showing MDR by age- and serotype group, Belgium 2018–2023 (n = 8,088 isolates)^a^

Age group in years	Non-PCV20				PCV13-related			PCV15-non-PCV13			PCV20-non-PCV15			All serotypes		
	Number resistant	Total	%		Number resistant	Total	%	Number resistant	Total	%	Number resistant	Total	%	Number resistant	Total	%
**Resistance to at least one antibiotic**																
< 2	132	246	53.7	70		224	31.3	32	61	52.5	39	156	25.0	273	687	39.7
2–15	95	165	57.6	52		158	32.9	4	19	21.1	20	135	14.8	171	477	35.8
16–65	214	659	32.5	270		1,162	23.2	38	202	18.8	160	1,179	13.6	682	3,202	21.3
> 65	321	1,055	30.4	414		1,424	29.1	71	315	22.5	163	924	17.6	969	3,718	26.1
**All ages**	**762**	**2,125**	**35.9**	**806**		**2,971^b^**	**27.1**	**145**	**597**	**24.3**	**382**	**2,395^c^**	**15.9**	**2,095**	**8,088^d^**	**25.9**
**MDR: resistance to three or more antibiotic groups (ATC level 3)**																
< 2	68	246	27.6	38		224	17.0	1	61	1.6	9	156	5.8	116	687	16.9
2–15	33	165	20.0	22		158	13.9	0	19	0.0	4	135	3.0	59	477	12.4
16–65	59	659	9.0	97		1,162	8.3	0	202	0.0	19	1,179	1.6	175	3,202	5.5
> 65	136	1,055	12.9	164		1,424	11.5	0	315	0.0	24	924	2.6	324	3,718	8.7
**All ages**	**296**	**2,125**	**13.9**	**321**		**2,971^b^**	**10.8**	**1**	**597**	**0.2**	**56**	**2,395^c^**	**2.3**	**674**	**8,088^d^**	**8.3**

ATC: anatomical therapeutic chemical classification; PCV: pneumococcal conjugate vaccine; MDR: multidrug resistance.

^a^ Among 8,123 isolates that were obtained between 2018 and 2023, 8,088 could be classified by serotype.

^b^ The number here (i.e. 2,971) is greater than the total of the column (2,968 = 224 + 158 + 1,162 + 1,424) because three patients with PCV13-related-serotype isolates were missing age information and had to be excluded from calculations by age group.

^c^ The number here (i.e. 2,395) is greater than the total of the column (2,394 = 156 + 135 + 1,179 + 924) because one patient with PCV20-non-PCV15 serotype isolates had missing age information and had to be excluded from calculations by age group.

^d^ The number here (i.e. 8,088) is greater than the total of the column (8,084 = 687 + 477 + 3,202 + 3,718) because in total four patients were missing age information and had to be excluded from calculations by age group.

Table 3 presents the prevalence of different groups of serotypes among the isolates. PCV13-related serotypes were most prevalent, whereas PCV15-non-PCV13 serotypes were least prevalent. Non-PCV20 serotypes accounted for 26.3% of the pneumococcal isolates, of which serotypes 9N, 23B and 15A were most common (271/2,125; 269/2,125 and 215/2,125, respectively) and serotypes 23B, 24F and 15A were most often resistant to at least one antibiotic (206/762; 179/762 and 62/762, respectively). PCV13-related serotypes accounted for 36.7% of the pneumococcal isolates, of which serotypes 3, 19A and 4 were most common (989/2,971; 973/2,971 and 333/2,971, respectively), and serotypes 19A, 6C and 19F were most often resistant to at least one antibiotic (302/806; 210/806 and 96/806, respectively). PCV15-non-PCV13 serotypes accounted for 7.4% of the pneumococcal isolates, of which serotype 22F was most common (387/597) and 33F was most often resistant to at least one antibiotic (130/145). PCV20-non-PCV15 serotypes accounted for 29.6% of all pneumococcal isolates, of which the most common serotypes were 8, 12F and 10A (1,310/2,395; 574/2,395 and 229/2,395, respectively). Serotypes 12F, 11A and 15B were most often resistant (163/382, 146/382 and 30/382, respectively). Table 4 shows the percentage of isolates resistant to at least one antibiotic and the percentage of isolates showing MDR by age- and serotype group. Among the non-PCV20 serotypes, 35.9% showed resistance to at least one antibiotic and 13.9% showed MDR. Among the PCV13-related serotypes, 27.1% showed resistance to at least one antibiotic and 10.8% showed MDR, i.e. resistance to three or more antibiotic groups (ATC level 3). Among the PCV15-non-PCV13 serotypes, 24.3% showed resistance to at least one antibiotic and 0.2% showed MDR. Among the PCV20-non-PCV15 serotypes, 15.9% showed resistance to at least one antibiotic and 2.3% showed MDR. Overall, 25.9% of all pneumococcal isolates showed resistance to at least one antibiotic and 8.3% showed MDR.

Table 5 shows the number and percentage of isolates resistant to penicillin, amoxicillin, cefotaxime, erythromycin, tetracycline and trimethoprim/sulfamethoxazole, as well as the number of isolates showing MDR, by serotype group. Pneumococcal isolates were mostly resistant to erythromycin and tetracycline (15.8% and 15.6%, respectively). In non-PCV20 serotypes, penicillin resistance was most common (23.9%). Erythromycin resistance was most common in PCV13-related serotypes (23.1%) and PCV15-non-PCV13 serotypes (22.6%). Tetracycline resistance was most common in PCV20-non-PCV15 serotypes (8.1%).

**Table 5:** Number and percentage of isolates resistant to penicillin, amoxicillin, cefotaxime, erythromycin, tetracycline and trimethoprim/sulfamethoxazole; and number of isolates showing multidrug resistance, by serotype group, Belgium 2018–2023 (n = 8,088 isolates)^a^

**Serotypes (number of isolates)**	**Antibiotic resistance**	**Number**	**%**
Non-PCV20 (n = 2,125)	Penicillin resistance	508	23.9
	Amoxicillin resistance	34	1.6
	Cefotaxime resistance	29	1.4
	Erythromycin resistance	398	18.7
	Tetracycline resistance	386	18.2
	Trimethoprim/sulfamethoxazole resistance	263	12.4
	MDR	296	13.9
PCV13-related (n = 2,971)	Penicillin resistance	386	13.0
	Amoxicillin resistance	140	4.7
	Cefotaxime resistance	110	3.7
	Erythromycin resistance	686	23.1
	Tetracycline resistance	596	20.1
	Trimethoprim/sulfamethoxazole resistance	142	4.8
	MDR	321	10.8
PCV15-non-PCV13 (n = 597)	Penicillin resistance	0	0.0
	Amoxicillin resistance	0	0.0
	Cefotaxime resistance	0	0.0
	Erythromycin resistance	135	22.6
	Tetracycline resistance	88	14.7
	Trimethoprim/sulfamethoxazole resistance	4	0.7
	MDR	1	0.2
PCV20-non-PCV15 (n = 2,395)	Penicillin resistance	148	6.2
	Amoxicillin resistance	55	2.3
	Cefotaxime resistance	44	1.8
	Erythromycin resistance	56	2.3
	Tetracycline resistance	194	8.1
	Trimethoprim/sulfamethoxazole resistance	177	7.4
	MDR	56	2.3
Total (n = 8,088)	Penicillin resistance	1,042	12.9
	Amoxicillin resistance	229	2.8
	Cefotaxime resistance	183	2.3
	Erythromycin resistance	1,275	15.8
	Tetracycline resistance	1,264	15.6
	Trimethoprim/sulfamethoxazole resistance	586	7.2
	MDR	674	8.3

ATC: anatomical therapeutic chemical classification; PCV: pneumococcal conjugate vaccine; MDR: multidrug resistance: resistance to three or more antibiotic groups (ATC level 3).

^a^ Among 8,123 isolates that were obtained between 2018 and 2023, 8,088 could be classified by serotype.

A strong correlation (> 0.5) was found between amoxicillin and cefotaxime on the one hand, and between erythromycin and tetracycline on the other hand (Table 6). Resistances to these antibiotics were therefore not simultaneously included as predictor variables, and separate multinomial regression models were fitted.

**Table 6:** Phi correlation coefficients for all antibiotic resistance pairs in *Streptococcus pneumoniae*, Belgium 2018–2023 (n = 8,123 isolates)

Antibiotic	Penicillin	Amoxicillin	Cefotaxime	Erythromycin	Tetracycline	Trimethoprim /sulfamethoxazole
Penicillin	1.00	0.44	0.39	0.46	0.39	0.41
Amoxicillin	0.44	1.00	**0.85**	0.25	0.18	0.25
Cefotaxime	0.39	**0.85**	1.00	0.21	0.14	0.26
Erythromycin	0.46	0.25	0.21	1.00	**0.75**	0.09
Tetracycline	0.39	0.18	0.14	**0.75**	1.00	0.12
Trimethoprim/sulfamethoxazole	0.41	0.25	0.26	0.09	0.12	1.00

Phi correlation coefficients above 0.5 were considered to indicate a strong correlation and are indicated in bold.

Table 7 shows the odds ratios (ORs) and corresponding 95% confidence intervals (95% CIs) according to the final model, describing the associations between antibiotic resistance patterns and serotype groups, while correcting for confounding variables. These results are based on the final model including resistance to amoxicillin and erythromycin and excluding resistance to cefotaxime and tetracycline as predictor variables. The results from the final models including cefotaxime and tetracycline as predictor variables, are shown in Supplementary Table S1 and Table S2, respectively.

**Table 7:** Odds ratios with 95% confidence intervals describing associations between antibiotic resistance and serotype groups, according to the final multinomial model including resistance to erythromycin and amoxicillin, correcting for confounders, Belgium, 2018–2023 (n = 8,088 isolates)^a^

Variable		Odds ratios of serotype groups (95% confidence intervals)		
		*P*(PCV13)/ *P*(non-PCV20)	*P*(PCV15-non-PCV13)/ *P*(non-PCV20)	*P*(PCV20-non-PCV15)/ *P*(non-PCV20)
**Amoxicillin**				
All ages	Susceptible	REF	NA	REF
	Resistant	7.65 (4.95–11.82)*	NA	9.45 (5.60–15.94)*
**Trimethoprim/sulfamethoxazole**				
All ages	Susceptible	REF	REF	REF
	Resistant	0.46 (0.36–0.60)*	0.11 (0.04–0.30)*	1.10 (0.85–1.43)
**Penicillin**				
< 2 years	Susceptible	REF	NA	REF
	Resistant	0.25 (0.14–0.44)*	NA	0.25 (0.13–0.47)*
2–15 years	Susceptible	REF	NA	REF
	Resistant	0.18 (0.10–0.34)*	NA	0.05 (0.02–0.13)*
16–65 years	Susceptible	REF	NA	REF
	Resistant	0.24 (0.17–0.34)*	NA	0.19 (0.12–0.28)*
> 65 years	Susceptible	REF	NA	REF
	Resistant	0.34 (0.25–0.46)*	NA	0.46 (0.31–0.68)*
**Erythromycin**				
< 2 years	Susceptible	REF	REF	REF
	Resistant	1.35 (0.78–2.31)	9.43 (4.71–18.88)*	0.18 (0.08–0.42)*
2–15 years	Susceptible	REF	REF	REF
	Resistant	2.10 (1.09–4.04)*	3.59 (1.00–12.86)	0.13 (0.03–0.58)*
16–65 years	Susceptible	REF	REF	REF
	Resistant	2.43 (1.74–3.40)*	3.69 (2.26–6.03)*	0.16 (0.10–0.28)*
> 65 years	Susceptible	REF	REF	REF
	Resistant	2.47 (1.92–3.19)*	3.61 (2.53–5.15)*	0.15 (0.10–0.24)*

NA: not applicable as no PCV15-non-PCV13 isolates were found to be resistant to penicillin or amoxicillin during the whole study period; PCV: pneumococcal conjugate vaccine; *P*(non-PCV20): probability of being a non-PCV20 serotype; *P*(PCV13): probability of being a PCV13-related serotype; *P*(PCV15-non-PCV13): probability of being a PCV15-non-PCV13 serotype; *P*(PCV20-non-PCV15): probability of being a PCV20-non-PCV15 serotype; REF: reference category in the multinomial model.

* Significant at 5% confidence level.

According to the model including resistance to amoxicillin and erythromycin (Table 7), PCV13-related serotypes and PCV20-non-PCV15 serotypes are more frequently associated with amoxicillin resistance compared with non-PCV20 serotypes (ORs: 7.65 and 9.45, respectively). Besides, PCV13-related serotypes and PCV15-non-PCV13 serotypes are less frequently associated with trimethoprim/sulfamethoxazole resistance compared with non-PCV20 serotypes (ORs: 0.46 and 0.11, respectively). It was further found that PCV13-related serotypes and PCV20-non-PCV15 serotypes are less frequently associated with penicillin resistance compared with non-PCV20 serotypes, where the strength of these associations differed between age groups (ORs: 0.18–0.34 and 0.05–0.46, respectively). The strength of the associations found between serotype groups and erythromycin resistance also differs between age groups. In individuals aged above 2 years, PCV13-related serotypes are more frequently associated with erythromycin resistance compared with non-PCV20 serotypes (ORs depending on age: 2.10–2.47). In individuals aged below 2 years and in individuals aged 16 years or above, PCV15-non-PCV13 serotypes are more frequently associated with erythromycin resistance compared with non-PCV20 serotypes (ORs depending on age: 3.59–9.43). Finally, PCV20-non-PCV15 serotypes are less frequently associated with erythromycin resistance compared with non-PCV20 serotypes (ORs depending on age: 0.13–0.18). Since no PCV15-non-PCV13 isolates were found to be resistant to penicillin or amoxicillin during the whole study period, the ORs for these categories could not be estimated.

According to the model including resistance to cefotaxime (Table S1), PCV13-related and PCV20-non-PCV15 serotypes are more frequently associated with cefotaxime resistance compared with non-PCV20 serotypes, where the strength of these associations differed between age groups (ORs depending on age: 3.26–67.82 and 5.06–82.38 respectively). Since no PCV15-non-PCV13 isolates were found to be resistant to cefotaxime during the whole study period, the OR for this category could not be estimated. According to the model including resistance to tetracycline (Table S2), PCV13-related serotypes and PCV15-non-PCV13 serotypes are more frequently associated with tetracycline resistance compared with non-PCV20 serotypes (ORs: 1.78 and 1.97, respectively). Finally, PCV20-non-PCV15 serotypes are less frequently associated with tetracycline resistance compared with non-PCV20 serotypes (OR: 0.71).

## Discussion

In this study we assessed the association between AMR and the additional non-PCV13 serotypes in PCV15 and PCV20, based on Belgian reference laboratory data of invasive *S. pneumoniae* from 2018 to 2023.

The annual numbers of IPD isolates almost halved during the COVID-19 pandemic in 2020 and 2021. In 2022, the number of IPD isolates returned to pre-pandemic levels, while it exceeded these levels in 2023 and 2024 [24,29]. As it is assumed that IPD surveillance remained stable during pandemic years, the decrease in IPD incidence during this time might be attributed to the implementation of non-pharmaceutical interventions to control COVID-19 [30,31]. This explanation is further supported by similar decreases in other countries [32,33]. As opposed to the decrease in IPD incidence, no decrease in pneumococcal carriage has been reported in infants attending daycare in Belgium between November 2020 and March 2021. This discordance between IPD incidence and pneumococcal carriage in infants indicates the possible importance of interspecies interactions in the risk of pneumococcal invasion [34]. It is further hypothesised that the increase in IPD isolates in 2023 was caused by an immunity decrease in people, post-COVID-19 pandemic [30,31,35]. Although the COVID-19 pandemic had a clear impact on the number of IPD cases and might have changed the pneumococcal serotype distribution, as was seen in the Netherlands [36], the annual age distribution of IPD in Belgium appeared to remain stable across the study period (data not shown) and we assume that influence of the pandemic on our analysis is limited. If certain serotypes are more prone to AMR due to intrinsic characteristics, it can be expected that this would still be the case during the COVID-19 pandemic, even if overall IPD incidence is lower or if the pneumococcal serotype distribution changed.

Clear differences in resistance patterns were observed between different age groups, where AMR was most frequent in individuals below the age of 2 years. These results are in accordance with previous studies that showed higher resistance rates in invasive *S. pneumoniae* isolated from younger age groups [37,38]. This observation can be explained by the overuse and misuse of antibiotics in these age groups, as it has been shown that the time since the last exposure to an antibiotic is the most important predicting factor for antibiotic resistance in pneumococci [39,40]. Clear differences in resistance patterns were also observed between different vaccine serotype groups, where AMR was most frequent in non-PCV20 serotypes and least frequent in PCV20-non-PCV15 serotypes.

A significant correlation was found between tetracycline and erythromycin resistance. This correlation has been observed before in *S. pneumoniae* and might be attributed to the Tn1545 transposon and related conjugative transposons, which encode erythromycin resistance via the *erm*(B) gene and tetracycline resistance via the *tet*(M) gene [41].

Our study further showed that, compared with non-PCV20 serotypes, PCV13-related serotypes are significantly more often associated with amoxicillin, cefotaxime, erythromycin and tetracycline resistance, and susceptibility to trimethoprim/sulfamethoxazole and penicillin. PCV15-non-PCV13 serotypes are significantly more often associated with erythromycin and tetracycline resistance, and with trimethoprim/sulfamethoxazole susceptibility, compared with non-PCV20 serotypes. At last, PCV20-non-PCV15 serotypes are significantly more often associated with resistance to amoxicillin and cefotaxime and susceptibility to penicillin, erythromycin and tetracycline compared with non-PCV20 serotypes. Associations between specific serotypes and the probability of resistance to specific antibiotics has been described before by Andrejko et al., who showed a higher prevalence of antibiotic resistance among serotypes with enhanced persistence in the upper respiratory tract and a high probability of causing otitis media and IPD [42]. The association between PCV13-related serotypes and resistance to cefotaxime and erythromycin has previously been observed by Savrasova et al. for serotypes 14, 19A and 19F. Conversely, the association identified in this study between penicillin resistance and non-PCV20 serotypes differs from the results obtained by Savrasova et al., who found an association between penicillin resistance and PCV10 serotypes [43]. However, it should be noted that the breakpoint used to determine penicillin resistance was different in our study.

As the PCV15-non-PCV13 serotypes accounted for only 7.4% of the IPD isolates in this study, it is expected that the implementation of PCV15 in the national immunisation programme will result in a rather small decrease in overall IPD incidence. Therefore, also the reduction in antibiotics used to treat IPD is expected to be limited. The PCV15-non-PCV13 serotypes were found to be correlated with increased resistance to erythromycin and tetracycline. Therefore, implementing PCV15 might directly lower AMR in pneumococci, on top of a potential reduction due to decreased antibiotic use. However, because of the low prevalence of PCV15-non-PCV13 serotypes, this effect is again expected to be limited.

The implementation of PCV20, on the other hand, could lead to a theoretical larger decrease in overall IPD incidence, as PCV20-non-PCV13 serotypes accounted for 37% of the IPD cases under study. Consequently, a larger reduction in the use of antibiotics to treat IPD could be expected, which in turn could lead to reduced resistance rates. Although PCV20 would be implemented in the vaccination schedule in infants younger than 2 years of age, a substantial decrease in IPD incidence can also be expected in the other age groups, due to population immunity [44]. However, as the PCV20-non-PCV15 serotypes were found to be mainly associated with antibiotic susceptibility, and with resistance to amoxicillin and cefotaxime which is rather uncommon, the additional impact of PCV20 vaccination on AMR in *S. pneumoniae* is likely to be limited, apart from the reduction in antibiotic use alone. Nevertheless, it should be noted that amoxicillin is a first-line antibiotic in the treatment of IPD. Therefore, the association between amoxicillin resistance and PCV20-non-PCV15 serotypes is still clinically relevant by avoiding this limited number of amoxicillin resistant cases, that would otherwise be hard to treat. Besides, the fact that PCV20-non-PCV15 serotypes are associated with higher levels of amoxicillin and cefotaxime resistance (MIC > 0.5 mg/L), and lower levels of penicillin resistance (MIC > 0.06 mg/L) compared with non-PCV20 serotypes, suggests that PCV20-non-PCV15 serotypes have higher MIC values and a higher level of β-lactam resistance compared with non-PCV20 serotypes, which mainly have a lower level of resistance.

Further, serotype replacement might lead to a relative increase in pneumococcal resistance rates, as our study showed a high percentage of penicillin, erythromycin and tetracycline resistant non-PCV20 serotypes, while the preventable PCV20-non-PCV15 serotypes were found to be associated with penicillin, erythromycin and tetracycline susceptibility. However, we can assume that the majority of non-PCV20 serotypes show a low level of β-lactam resistance (penicillin MIC > 0.06 mg/L, amoxicillin MIC < 0.5 mg/L) compared with the PCV20-non-PCV15 serotypes and might still be treated with a higher dose of penicillin or with broader spectrum β-lactams (e.g. cefotaxime). Our results are consistent with those of previous studies, which showed that the health and economic benefits of PCV20 vaccination are substantially greater than those of PCV15 vaccination [45-49]. Nevertheless, close monitoring remains recommended, especially given the high percentage of resistant non-PCV20 serotypes found in our study and the potential for serotype replacement.

To the best of our knowledge, this is the first study that investigated the association between antibiotic resistance patterns and pneumococcal vaccine serotype groups in Belgium, while correcting for confounding variables using multinomial logistic regression models. To avoid overcomplicating the model, only variables assumed to have an impact on serotype were included. Sex at birth was therefore not considered. Since previous studies showed no sex differences in pneumococcal serotype distribution, we believe this does not have a major impact on the study results [50]. Information on some other demographic key variables such as race and/or ethnicity was lacking, which could potentially influence our results. Furthermore, as the vaccination status of the individuals contributing to this study was unknown, we were unable to correct for this variable. However, the model implicitly accounts for vaccination status, since the age categories used in the model were based on those used for vaccination in Belgium. As more than 92% of Belgian laboratories contributed to the IPD isolates analysed in our study, we are confident that there is no selection bias and that our results accurately reflect the true epidemiology of pneumococcal disease in Belgium. Although our study obtained reliable estimates on these associations using a large sample size, the model did not allow to evaluate which specific serotypes contributed most to these associations. Besides, extrapolating these results to other countries should be done with care, due to potential differences in pneumococcal epidemiology, vaccine policies and antibiotic consumption levels. Nevertheless, our findings are important for public health and can be used to inform national vaccination strategies in Belgium and other countries with similar characteristics. Besides, the modelling strategy applied in our study can also be adopted in other settings and countries. As the analyses were performed on data from IPD, the associations found between antibiotic resistance patterns and serotype groups might also not hold for non-invasive pneumococcal diseases, which are associated with different serotypes than invasive infections, and where antibiotic resistance is even more prevalent [51].

## Conclusion

In conclusion, based on retrospective reference laboratory data from 2018 through 2023, implementation of PCV15 in the national immunisation programme in Belgium is expected to have a limited impact on IPD incidence and pneumococcal AMR levels. The implementation of PCV20, on the other hand, may lead to a substantial decrease in overall IPD incidence, and consequently in the use of antibiotics to treat IPD. As the PCV20-non-PCV15 serotypes are associated with increased resistance to the first-line antibiotic amoxicillin, a limited number of resistant and otherwise hard to treat infections could be avoided. Although serotype replacement might lead to a relative increase in isolates with a low level of penicillin resistance, the majority of these infections should still be treatable with a higher concentration of penicillin or broader spectrum β-lactams. These findings are important for public health and can be used to inform national vaccination strategies in Belgium and other countries with similar characteristics. In any case, close monitoring remains recommended.

## Data Availability

The data that support this study were obtained as part of the routine surveillance of the National Reference Centre for invasive *S. pneumoniae* UZ Leuven (as defined by the Royal Decree of 09/02/2011). Restrictions apply to the availability of these data, which were used under licence for this study. A data transfer agreement was registered with the Data Protection Board of UZ Leuven (dp024).

## Supplementary Data

44000 SupplementaryMaterial
