# Supplementary material for: Higher valency vaccines’ impact on antimicrobial resistance rates in Streptococcus pneumoniae causing invasive disease: a retrospective analysis based on national reference laboratory data, Belgium, 2018 to 2023
Source: Euro Surveill. 2025 Nov 13;30(45):2500179. doi: 10.2807/1560-7917.ES.2025.30.45.2500179 (PMC12633709; doi:10.2807/1560-7917.ES.2025.30.45.2500179)
Supplement: SupplementaryMaterial [file 25-00179_SupplementaryMaterial.pdf]

Supplementary files for article: "Higher valency vaccines' impact on antimicrobial resistance rates in *Streptococcus pneumoniae* causing invasive disease: a retrospective analysis based on national reference laboratory data, Belgium, 2018 to 2023".

This supplementary material is hosted by Eurosurveillance as supporting information alongside the article *"Higher valency vaccines' impact on antimicrobial resistance rates in Streptococcus pneumoniae causing invasive disease: a retrospective analysis based on national reference laboratory data, Belgium, 2018 to 2023"*, on behalf of the authors, who remain responsible for the accuracy and appropriateness of the content. The same standards for ethics, copyright, attributions and permissions as for the article apply. Supplements are not edited by Eurosurveillance and the journal is not responsible for the maintenance of any links or email addresses provided therein.

Table S1: Odds ratio's (ORs) and corresponding 95% confidence intervals (95% CIs) according to the multinomial model including resistance to cefotaxime, describing the associations between antibiotic resistance and serotype groups, correcting for confounders, Belgium 2018-2023.

		$\frac{P(PCV13)}{P(Non-PCV20)}$	$\frac{P(PCV15-non-PCV13)}{P(Non-PCV20)}$	$\frac{P(PCV20-non-PCV15)}{P(Non-PCV20)}$
**Trimethoprim/sulfamethoxazole**				
**All ages**	**Susceptible**	REF	REF	REF
	**Resistant**	0.44 (0.34 ; 0.57)*	0.10 (0.04 ; 0.29)*	1.09 (0.84 ; 1.41)
**Penicillin**				
**<2 years**	**Susceptible**	REF	NA	REF
	**Resistant**	0.19 (0.10 ; 0.37)*	NA	0.24 (0.07 ; 8.15)
**2-15 years**	**Susceptible**	REF	NA	REF
	**Resistant**	0.17 (0.09 ; 0.32)*	NA	0.04 (0.01 ; 0.12)*
**16-65 years**	**Susceptible**	REF	NA	REF
	**Resistant**	0.29 (0.20 ; 0.42)*	NA	0.21 (0.14 ; 0.32)*
**>65 years**	**Susceptible**	REF	NA	REF
	**Resistant**	0.39 (0.29 ; 0.54)*	NA	0.56 (0.38 ; 0.82)*
**Cefotaxime**				
**<2 years**	**Susceptible**	REF	NA	REF
	**Resistant**	67.82 (7.58 ; 607.19)*	NA	25.71 (1.92 ; 344.15)*
**2-15 years**	**Susceptible**	REF	NA	REF
	**Resistant**	48.91 (5.46 ; 437.86)*	NA	82.38 (6.15 ; 1103.05)*
**16-65 years**	**Susceptible**	REF	NA	REF
	**Resistant**	3.26 (1.59 ; 6.69)*	NA	5.29 (2.15 ; 12.99)*
**>65 years**	**Susceptible**	REF	NA	REF
	**Resistant**	4.90 (2.47 ; 9.75)*	NA	5.06 (2.25 ; 11.39)*
**Erythromycin**				
**<2 years**	**Susceptible**	REF	REF	REF
	**Resistant**	1.37 (0.70 ; 2.71)	9.63 (2.66 ; 34.80)*	0.21 (0.04 ; 0.95)*
**2-15 years**	**Susceptible**	REF	REF	REF
	**Resistant**	2.20 (1.12 ; 4.34)*	3.72 (1.03 ; 13.44)*	0.13 (0.03 ; 0.62)*
**16-65 years**	**Susceptible**	REF	REF	REF
	**Resistant**	2.55 (1.82 ; 3.56)*	3.84 (2.35 ; 6.27)*	0.17 (0.10 ; 0.29)*
**>65 years**	**Susceptible**	REF	REF	REF
	**Resistant**	2.50 (1.94 ; 3.22)*	3.66 (2.57 ; 5.22)*	0.16 (0.10 ; 0.25)*

P(PCV13) = probability of being a PCV13-related serotype; ***P(PCV15-non-PCV13)*** = probability of being a PCV15-non-PCV13 serotype; ***P(PCV20-non-PCV15)*** = probability of being a PCV20-non-PCV15 serotype; ***P(non-PCV20)*** = probability of being a non-PCV20 serotype; **NA** = not applicable; **PCV** = pneumococcal conjugate vaccine; **REF** = reference category in the multinomial model; ***** = significant at 5% confidence level.

Table S2: Odds ratio's (ORs) and corresponding 95% confidence intervals (95% CIs) according to the multinomial model including resistance to tetracycline, describing the associations between antibiotic resistance and serotype groups, correcting for confounders, 2018-2023.

		$\frac{P(PCV13)}{P(Non-PCV20)}$	$\frac{P(PCV15-non-PCV13)}{P(Non-PCV20)}$	$\frac{P(PCV20-non-PCV15)}{P(Non-PCV20)}$
**Amoxicillin**				
**All ages**	**Susceptible**	REF	NA	REF
	**Resistant**	8.61 (5.60 ; 13.24)*	NA	7.18 (4.37 ; 11.78)*
**Tetracycline**				
**All ages**	**Susceptible**	REF	REF	REF
	**Resistant**	1.78 (1.50 ; 2.12)*	1.97 (1.50 ; 2.59)*	0.71 (0.58 ; 0.87)*
**Penicillin**				
**<2 years**	**Susceptible**	REF	NA	REF
	**Resistant**	0.18 (0.11 ; 0.29)*	NA	0.12 (0.06 ; 0.24)*
**2-15 years**	**Susceptible**	REF	NA	REF
	**Resistant**	0.19 (0.11 ; 0.33)*	NA	0.05 (0.02 ; 0.14)*
**16-65 years**	**Susceptible**	REF	NA	REF
	**Resistant**	0.30 (0.21 ; 0.42)*	NA	0.12 (0.08 ; 0.18)*
**>65 years**	**Susceptible**	REF	NA	REF
	**Resistant**	0.51 (0.38 ; 0.67)*	NA	0.28 (0.19 ; 0.40)*
**Trimethoprim/sulfamethoxazole**				
**<2 years**	**Susceptible**	REF	REF	REF
	**Resistant**	1.07 (0.55 ; 2.07)	0.85 (0.18 ; 4.07)	2.11 (1.02 ; 4.38)*
**2-15 years**	**Susceptible**	REF	NA	REF
	**Resistant**	0.56 (0.26 ; 1.20)	NA	0.49 (0.17 ; 1.36)
**16-65 years**	**Susceptible**	REF	REF	REF
	**Resistant**	0.51 (0.33 ; 0.80)*	0.15 (0.02 ; 1.12)	1.37 (0.89 ; 2.09)
**>65 years**	**Susceptible**	REF	REF	REF
	**Resistant**	0.28 (0.19 ; 0.41)*	0.06 (0.01 ; 0.44)*	1.16 (0.81 ; 1.66)

P(PCV13) = probability of being a PCV13-related serotype; ***P(PCV15-non-PCV13)*** = probability of being a PCV15-non-PCV13 serotype; ***P(PCV20-non-PCV15)*** = probability of being a PCV20-non-PCV15 serotype; ***P(non-PCV20)*** = probability of being a non-PCV20 serotype; **NA** = not applicable; **PCV** = pneumococcal conjugate vaccine; **REF** = reference category in the multinomial model; ***** = significant at 5% confidence level.
